# Surgical treatment and prognosis in patients with intestinal metastases originated from advanced epithelial ovarian cancer

**DOI:** 10.3389/fonc.2026.1760552

**Published:** 2026-06-15

**Authors:** Hongxia Wang, Yijie Li, Zhifen Yang, Jinxiu Wang, Kaiyun Qin, Yu Yu, Na Wang, Jingde Jia, Wenhong Zhao, Fenghua Zhang, Mario M. Leitao, Ran Meng, Yueping Liu, Yan Ding, Zhengmao Zhang

**Affiliations:** 1 Department of Gynecology, Fourth Hospital of Hebei Medical University, Shijiazhuang, Hebei, China; 2 Department of Obstetrics, Fourth Hospital of Hebei Medical University, Shijiazhuang, Hebei, China; 3 Department of Gynecology, Hebei General Hospital, Shijiazhuang, Hebei, China; 4 Department of General Surgery, Hebei General Hospital, Shijiazhuang, Hebei, China; 5 Gynecology Service, Department of Surgery, Memorial Sloan Kettering Cancer Center, New York, NY, United States; 6 Department of Pathology, Fourth Hospital of Hebei Medical University, Shijiazhuang, Hebei, China

**Keywords:** advanced ovarian cancer, intestinal metastasis, intestinal resection, intestinal tumor stripping, prognosis, surgical treatment

## Abstract

**Objective:**

To analyze the surgical treatment and prognosis of patients with advanced epithelial ovarian cancer with intestinal metastases.

**Methods:**

A retrospective study was conducted on 255 patients diagnosed with epithelial ovarian cancer stage III or IV with bowel metastases between January 1st, 2015 and December 31st, 2020, divided into two groups, the study group was the bowel resection group (101 cases), and the control group was the bowel tumor stripping group (154 cases).

**Results:**

R0 rate reached 75.4% in this cohort study. Patients in the bowel resection group were more likely to be in stage IV and had higher surgical complexity scores (*P=*0.021). The incidence of intraoperative blood transfusion, pneumonia and pleural effusion was significantly lower in the bowel tumor stripping group than in the bowel resection group. The rate of anastomotic fistula was 1.98% in the bowel resection group and 0% in the tumor-stripping group. It was similar between groups in terms of 5-year overall survival (OS) and 5-year progression free survival (PFS), 65.8% vs 73.8%, 70.6% vs 80.3%. 5-year OS was similar between the tumor stripping group with only residual lesions in the bowel wall and the bowel resection group, but 5-year PFS was significantly higher in the bowel resection group (*P=*0.032). No matter what PDS or IDS was used, 5-year OS and 5-year PFS were similar in both groups after cytoreductive surgery reached R0.

**Conclusion:**

Bowel tumor stripping does not negatively affect the prognosis of advanced ovarian cancer if R0 cytoreductive surgery is achieved. Excessive bowel resection does not improve overall survival.

## Introduction

Maximal cytoreductive surgery plays a critical role in the management of advanced ovarian cancer. Because ovarian cancer is typically diagnosed at an advanced stage and spreads predominantly via peritoneal implantation, the small and large intestines are frequently involved. Consequently, intestinal surgery has always been an integral component of debulking procedures (1, 2). Complete resection of tumor lesions is a major prognostic factor for patients with advanced ovarian cancer (3, 4). Among the intestinal metastatic sites, the rectosigmoid colon is the most commonly affected (in 24-64% of patients undergoing surgery for ovarian cancer), followed by the ileocecum (5, 6).

Several studies have addressed the intraoperative management of intestinal metastases in ovarian cancer. The choice of procedure depends on the size and depth of invasion of the lesion within the intestinal wall. The goal is to achieve no macroscopic residual disease or residual lesions smaller than 1 cm. The preferred approach is maximal tumor resection while preserving bowel integrity. The extent of bowel involvement is often difficult to predict preoperatively; therefore, preoperative bowel preparation is recommended, and patients or their families should be counseled regarding the possibility and necessity of bowel resection and potential stoma creation. If intraoperative findings reveal extensive infiltration of the deep muscular layer, intestinal resection should be performed promptly to avoid postoperative complications such as perforation, acute peritonitis, fistula, or obstruction.

Comparative analyses of different surgical techniques are essential. Some studies suggest that tumor stripping, compared with rectosigmoid resection, may result in microscopic residual disease that affects survival (7, 8); however, others indicate that tumor stripping of the rectosigmoid is not inferior to low anterior resection in terms of oncologic outcomes (9–11). Regardless of the specific approach, the completeness of cytoreduction remains a key determinant of prognosis in patients with advanced ovarian cancer. To date, no studies have clarified the factors linking colorectal resection to prognosis. The surgical strategy must be individualized based on intraoperative findings and the patient’s overall condition and needs. Therapeutic outcomes for cancer patients should encompass minimizing postoperative complications and preserving quality of life—a primary focus of gynecologic oncologists.

The present study aimed to analyze the surgical management and prognosis of patients with advanced epithelial ovarian cancer and intestinal metastases, specifically investigating postoperative complications, the impact of neoadjuvant chemotherapy (NACT) on the rate of bowel resection, and the effect of extensive bowel resection on prognosis.

## Materials and methods

### Patients

A total of 255 patients with pathologically confirmed intestinal metastases were selected from those with International Federation of Gynecology and Obstetrics (FIGO) stage III or IV primary epithelial ovarian cancer who underwent surgical treatment at the Fourth Hospital of Hebei Medical University between January 2015 and December 2020. They were divided into two groups according to the surgical procedure: the intestinal resection group (study group, n = 101) and the intestinal tumor stripping group (control group, n = 154). Patients’ baseline characteristics, surgical records, postoperative outcomes, and other relevant information were collected from medical records and follow-up data. Surgical stage and pathological grade were determined according to the FIGO criteria.

Inclusion criteria were: primary epithelial ovarian cancer; initial comprehensive staging cytoreductive surgery via laparotomy achieving R0 (no macroscopic residual disease) or R1 (residual lesions <1 cm in diameter); FIGO stage III or IV; intraoperative bowel resection or stripping of metastatic bowel tumors; and postoperative pathological confirmation of intestinal metastases. Exclusion criteria were: recurrent ovarian cancer treated by surgery; minimally invasive surgery; cytoreductive surgery with residual lesions >1 cm (R2); FIGO stage I or II; no bowel resection or tumor stripping performed; non−primary or non−epithelial ovarian cancer; and absence of intestinal metastases on postoperative pathology. Patients who met the inclusion criteria but had incomplete follow-up information (missed or refused visits) were also excluded.

All patients underwent laparotomy, total hysterectomy, bilateral salpingo−oophorectomy, and omentectomy. Appendectomy was performed if tumor infiltration was present or mucinous ovarian cancer was suspected. If enlarged or suspicious lymph nodes were found intraoperatively, lymphadenectomy was typically performed. In cases with no lymph node abnormalities, the decision to perform lymph node dissection was left to the attending physician’s discretion. Additional organ resections were performed as necessary depending on tumor invasion.

The surgical strategy for intestinal metastases was determined based on intraoperative assessment of the depth of tumor invasion. If the tumor did not invade the seromuscular layer, tumor stripping was performed, leaving the bowel wall intact without need for repair. If the tumor involved only the seromuscular layer, intestinal tumor stripping was performed: at the involved segment, the tumor together with a 5−mm margin of the underlying seromuscular tissue was excised. The resulting defect in the bowel wall was closed with interrupted 3−0 absorbable sutures using a seromuscular burying technique. If the tumor invaded through the muscularis propria or demonstrated full−thickness penetration, segmental bowel resection was performed directly.

All patients received a liquid diet starting 3 days before surgery for bowel preparation and took oral laxatives 1 day preoperatively. According to current guidelines, NACT was indicated for patients with advanced epithelial ovarian cancer who were not candidates for immediate surgery due to extensive disease and high perioperative risk. NACT consisted of platinum−based combination chemotherapy, typically paclitaxel plus carboplatin, administered every 3 weeks for 4 cycles. Patients who underwent interval debulking surgery (IDS) and achieved R0 resection received at least 3 cycles of adjuvant chemotherapy postoperatively. Patients who underwent primary debulking surgery (PDS) received postoperative platinum−based first−line chemotherapy, either alone or in combination with paclitaxel, every 3-4 weeks for 6-8 cycles. The follow−up period for all patients ranged from 2 to 98 months.

### Observation indices

Overall survival (OS) was defined as the time from the date of surgery to death from any cause or the last follow−up. Progression−free survival (PFS) was defined as the time from the date of surgery to subsequent disease progression or recurrence. The primary outcome was OS in patients undergoing bowel resection versus tumor stripping. Secondary outcomes included PFS, as well as 1−, 3−, and 5−year survival rates. Additional observations included whether the number of neoadjuvant chemotherapy cycles affected the rate of bowel resection and the occurrence of perioperative complications.

### Statistical analysis

Statistical analyses were performed using SPSS software (version 22.0). Continuous data conforming to a normal distribution were expressed as mean ± standard deviation (SD). Between−group comparisons were conducted using the t−test, Pearson’s chi−square test, or Fisher’s exact test, as appropriate. Differences in OS and PFS between groups were assessed using Kaplan−Meier survival analysis with the log−rank test. The association between the number of neoadjuvant chemotherapy cycles and the rate of bowel resection was analyzed using a logistic regression model. A two−sided p−value < 0.05 was considered statistically significant.

## Results

### Basic characteristics of patients

There were no significant differences in median age or histopathological type between patients who underwent bowel resection and those who underwent tumor stripping (**Table 1**). A higher proportion of patients with FIGO stage IV disease was observed in the bowel resection group (*P* = 0.021). The median length of hospital stay was significantly longer in the bowel resection group than in the tumor stripping group (*P* = 0.001). No statistically significant differences were found between the two groups with respect to preoperative administration of neoadjuvant chemotherapy (*P* = 0.298) or the number of neoadjuvant chemotherapy cycles (*P* = 0.521) (**Table 1**). In a logistic regression model, the odds ratio for bowel resection in patients receiving four or more cycles of neoadjuvant chemotherapy was 1.402 (*P* = 0.991) compared with those receiving three or fewer cycles.

**Table 1:** Preoperative basic characteristic data of ovarian cancer patients.

Characteristics	Bowel Resection group(n=101)	Bowel Tumor Stripping group (n=154)	*P* value
Age (y)	56.24 ± 9.51	54.90 ± 9.15	0.261
FIGO Stage (2014)			0.021*
IIIA	5 (4.95)	10 (6.49)	
IIIB	5 (4.95)	20 (12.99)	
IIIC	70 (69.31)	108 (70.13)	
IV	21 (20.79)	16 (10.39)	
Pathology			0.097
Endometrioid	4 (4.0)	18 (11.7)	
Mucinous	1 (1.0)	2 (1.3)	
Serous	88 (87.1)	114 (74.0)	
High grade	86 (97.73)	109 (95.61)	
Low grade	2 (2.27)	5 (4.39)	
Clear Cell	0 (0)	1 (0.6)	
Mixed	2 (2.0)	6 (3.1)	
Serous Cystadenocarcinoma	3 (3.0)	2 (2.0)	
Others	3 (3.0)	11 (7.1)	
Hospital Stays (d)	22.77 ± 9.54	19.40 ± 5.77	0.001*
Surgical Type			0.298
PDS	75 (74.26)	105 (68.18)	
IDS	26 (25.74)	49 (31.82)	
NACT Cycles			0.521
1-3	19 (73.08)	39 (79.59)	
≥4	7 (26.92)	10 (20.41)	

* P < 0.05, indicating a statistically significant difference.

In the bowel resection group, 80 patients (79.21%) had FIGO stage III disease and 21 (20.79%) had stage IV disease. In the tumor stripping group, 138 patients (89.61%) had stage III disease and 16 (10.39%) had stage IV disease. Regarding histopathological types in the bowel resection group, serous carcinoma was identified in 88 patients (87.1%), among whom 86 had high−grade and 2 had low−grade serous carcinoma; endometrioid carcinoma in 4 (4.0%); mucinous carcinoma in 1 (1.0%); mixed carcinoma in 2 (2.0%); serous cystadenocarcinoma in 3 (3.0%); and other pathologic types in 3 (3.0%). In the tumor stripping group, serous carcinoma was identified in 114 patients (74.0%), including 109 with high−grade and 5 with low−grade serous carcinoma; endometrioid carcinoma in 18 (11.7%); mucinous carcinoma in 2 (1.3%); clear−cell carcinoma in 1 (0.6%); mixed carcinoma in 6 (3.1%); serous cystadenocarcinoma in 2 (1.3%); and other pathologic types in 11 (7.1%). PDS was performed in 75 patients (74.26%) in the bowel resection group and in 105 patients (68.18%) in the tumor stripping group; IDS was performed in 26 (25.74%) and 49 (31.82%) patients, respectively (**Table 1**).

### Surgical characteristics of patients

Detailed information regarding surgical procedures, surgical outcomes, and the administration of postoperative adjuvant chemotherapy for patients in the two groups is presented in **Table 2**. The surgical complexity score (SCS) is a standardized quantitative scoring system to assess the extent, technical difficulty, and invasiveness of cytoreductive surgery for advanced ovarian cancer, with higher scores indicating more extensive resection and greater complexity. Patients in the bowel resection group had a significantly higher SCS than those in the tumor stripping group (*P* < 0.001).In the bowel resection group, 81 patients (80.20%) underwent intestinal anastomosis, of whom 63 (62.38%) received rectal anastomosis. Among these, 77 patients (95.06%) had a single intestinal anastomosis, and 4 patients (4.94%) had two intestinal anastomoses. A permanent or protective stoma was created in 27 patients (26.73%) in the bowel resection group. A higher proportion of patients in the bowel resection group underwent upper abdominal surgery, including subphrenic peritonectomy (*P* = 0.001) and splenectomy (*P* = 0.016), compared with the tumor stripping group. The incidence of lymphadenectomy (pelvic/para−aortic) or partial hepatectomy did not differ significantly between the two groups.

**Table 2:** Surgical characteristics of ovarian cancer patients.

	Bowel resection group (n=101)	Bowel tumor stripping group (n=154)	*P* value
SCS	7.46 ± 1.93	4.22 ± 1.22	<0.001*
Rectal Anastomosis	63 (62.38)	0	
No. of Intestinal Anastomosis	81 (80.20)		
1	77 (95.06)		
2	4 (4.94)		
Stoma	27 (26.73)		
Lymphadenectomy (Pelvic+/-Para-aortic)	67 (66.34)	86 (55.84)	0.094
Subphrenic Peritonectomy	21 (20.79)	10 (6.49)	0.001*
Splenenctomy	8 (7.92)	2 (1.30)	0.016*
Partial Hepatectomy	1 (0.99)	1 (0.65)	1.0
Partial Pancreatectomy	2	0	
Partial Cystectomy	0	1	
Residual Disease			0.256
R0	81 (80.20)	114 (74.03)	
R1(<1cm)	20 (19.80)	40 (25.97)	
Chemotherapy			1.0
Yes	98 (97.03)	149 (96.75)	
No	3 (2.97)	5 (3.25)	

* P < 0.05, indicating a statistically significant difference.

The surgical goal was to achieve R0 or R1 cytoreduction. R0 resection was achieved in 81 patients (80.20%) in the bowel resection group and 114 patients (74.03%) in the tumor stripping group; R1 resection was achieved in 20 patients (19.80%) and 40 patients (25.97%), respectively. The vast majority of patients continued to receive adjuvant chemotherapy: 98 patients (97.03%) in the bowel resection group and 149 patients (96.75%) in the tumor stripping group. The rate of adjuvant chemotherapy did not differ significantly between the two groups (*P* = 1.00) (**Table 2**).

Among the 101 patients in the bowel resection group, 84 (83.17%) underwent resection of a single bowel segment. This group included 47 patients who underwent low anterior resection (LAR), 21 patients who underwent partial rectal resection, 3 patients who underwent sigmoid colon resection, 3 patients who underwent partial colectomy, and 17 patients who underwent partial small bowel resection. A total of 17 patients (16.83%) underwent multisegmental bowel resection, including: LAR with left hemicolectomy (n = 3), LAR with right hemicolectomy (n = 2), LAR with partial colectomy (n = 1), LAR with ileocecal resection (n = 1), LAR with small bowel resection (n = 3), partial rectal resection with left hemicolectomy (n = 2), partial rectal resection with right hemicolectomy (n = 2), partial rectal resection with ileocecal resection (n = 1), total colectomy (n = 1), and subtotal colectomy (n = 1) (**Table 3**).

**Table 3:** Types of bowel resection.

Multi-segment bowel resection	n=17
LAR+left hemicolectomy	3
LAR+right hemicolectomy	2
LAR+partial colectomy	1
LAR+ ileocecal resection	1
LAR+small bowel resection	3
Partial rectal resection+left hemicolectomy	2
Partial rectal resection+right hemicolectomy	2
Partial rectal+ileocecal resection	1
Total colectomy	1
Subtotal colectomy	1
Single-segment bowel resection	n=84
LAR	47
Partial rectal resection	21
Sigmoid colon resection	3
Partial colectomy	3
Partial small bowel resection	17

### Postoperative complications

Postoperative complications occurred in 55 patients (54.46%) in the bowel resection group and in 80 patients (51.95%) in the tumor stripping group. Although the incidence of perioperative surgical complications appeared higher in the bowel resection group, the difference between the two groups was not statistically significant (*P* = 0.695). In separate analyses of specific postoperative adverse events, the rates of lower limb edema, lymphocele, wound infection, pulmonary atelectasis, lower extremity thrombosis, urinary tract infection, obturator nerve injury, and incisional hernia did not differ significantly between the two groups. However, the rate of intraoperative red blood cell transfusion was significantly higher in the bowel resection group than in the tumor stripping group (*P* = 0.005). The incidences of pneumonia (*P* = 0.001) and pleural effusion (*P* < 0.001) were also significantly higher in the bowel resection group. In the bowel resection group, postoperative complications included anastomotic bleeding (n = 2), anastomotic fistula (n = 2), secondary fistula (n = 1), intestinal obstruction (n = 19), acute kidney injury (n = 1), and upper gastrointestinal hemorrhage (n = 1). In the tumor stripping group, postoperative complications included pulmonary embolism (n = 1), diaphragmatic hernia (n = 1), and umbilical hernia (n = 1) (**Table 4**).

**Table 4:** Postoperative complications of patients.

	Bowel resection group (n=101)	Bowel tumor stripping group (n=154)	χ^2^	*P* value
Postoperative complications			0.695	0.695
Yes	55 (54.46)	80 (51.95)		
No	46 (45.54)	74 (48.05)		
Intraoperative Red Blood Cell Infusion			7.978	0.005*
Yes	77 (76.24)	91 (59.09)		
No	24 (23.76)	63 (40.90)		
No. of red blood cells transfused intraoperatively	3.88 ± 3.06	2.37 ± 2.47		<0.001*
lymphocele	17 (16.83)	34 (22.08)	1.049	0.306
Lower Limb Edema	6 (5.94)	13 (8.44)	0.553	0.457
Wound Infection	3 (2.97)	2 (1.30)		0.388
Pneumonia	13 (12.87)	4 (2.60)	10.347	0.001*
Pleural Effusion	24 (23.76)	8 (5.19)	19.162	<0.001*
Pulmonary Atelectasis	3 (2.97)	3 (1.95)		0.684
Lower Extremity Thrombosis	6 (5.94)	9 (5.84)	0.001	0.974
Urinary Tract Infection	3 (2.97)	4 (2.60)		1.000
Obturator Nerve Injury	9 (8.91)	11 (7.14)	0.264	0.608
Incisional Hernia	4 (3.96)	1 (0.65)		0.160
Others	Anastomotic Bleeding 2 Anastomotic Fistula 2 Secondary Fistula 1 Intestinal Obstruction 19 Acute kidney Injury 1 Upper Gastrointestinal Hemorrhage 1	Pulmonary Embolism 1 Diaphragmatic Hernia 1 Umbilical Hernia 1		

* P < 0.05, indicating a statistically significant difference.

### Survival analysis

**Figure 1:**
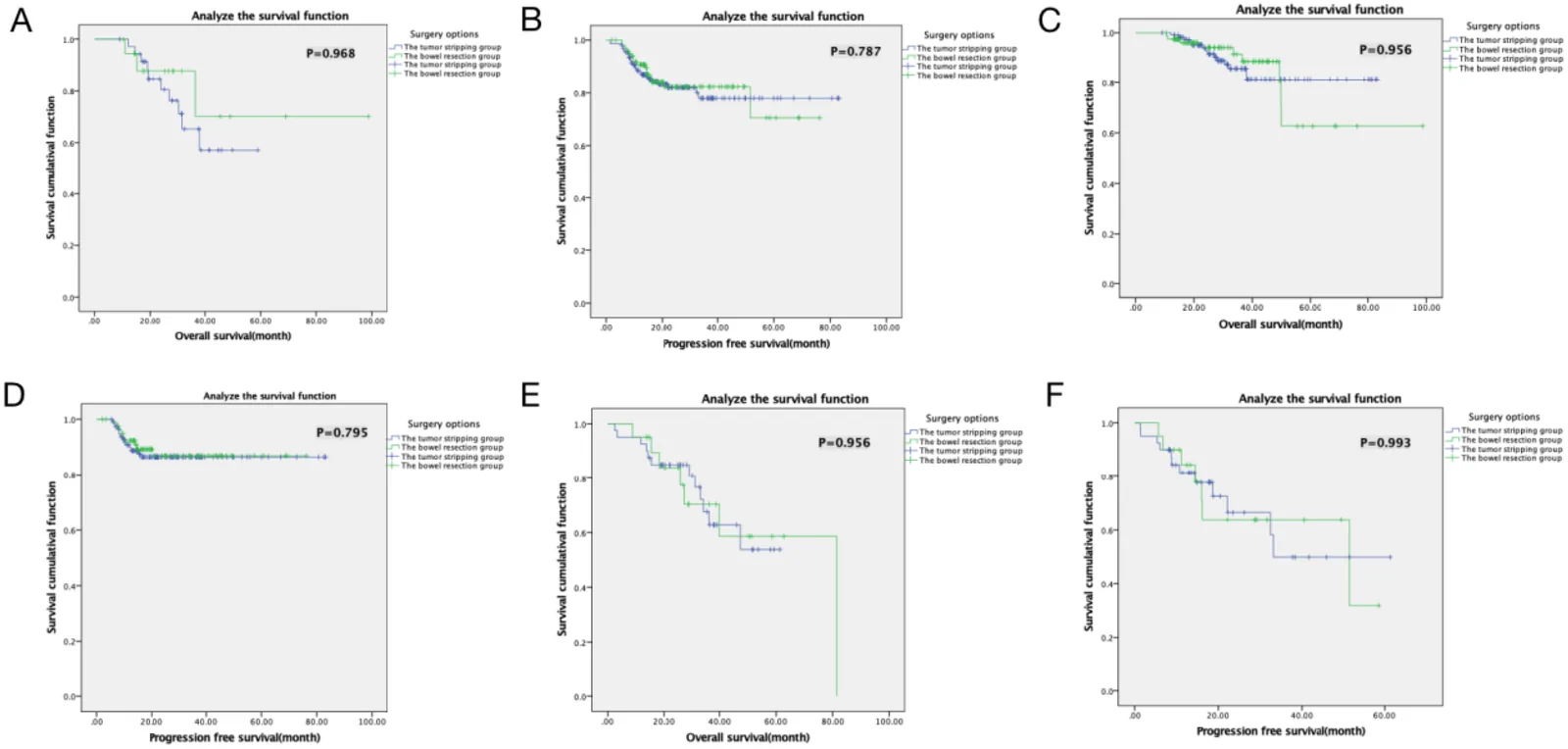
**(A)** Comparison of overall survival between the bowel resection group and the tumor stripping group. **(B)** Comparison of progression free survival between the bowel resection group and the tumor stripping group. **(C)** Comparison of overall survival between the bowel resection group and the tumor stripping group in R0 patients. **(D)** Comparison of progression free survival between the bowel resection group and the tumor stripping group in R0 patients. **(E)** Comparison of overall survival between the bowel resection group and the tumor stripping group in R1 patients. **(F)** Comparison of progression free survival between the bowel resection group and the tumor stripping group in R1 patients.

The follow−up period for the study ranged from 2 to 98 months. OS did not differ significantly between the bowel resection and tumor stripping groups (*P* = 0.968), nor did PFS (*P* = 0.787) (**Figures 1A, B**). R0 resection was achieved in 81 patients in the bowel resection group and in 114 patients in the tumor stripping group. Among these R0 patients, OS (*P* = 0.956) and PFS (*P* = 0.795) were not significantly different between the two groups (**Figures 1C, D**). R1 resection was achieved in 20 patients in the bowel resection group and in 40 patients in the tumor stripping group. Among these R1 patients, OS (*P* = 0.956) and PFS (*P* = 0.993) also showed no significant differences between the two groups (**Figures 1E, F**).

**Figure 2:**
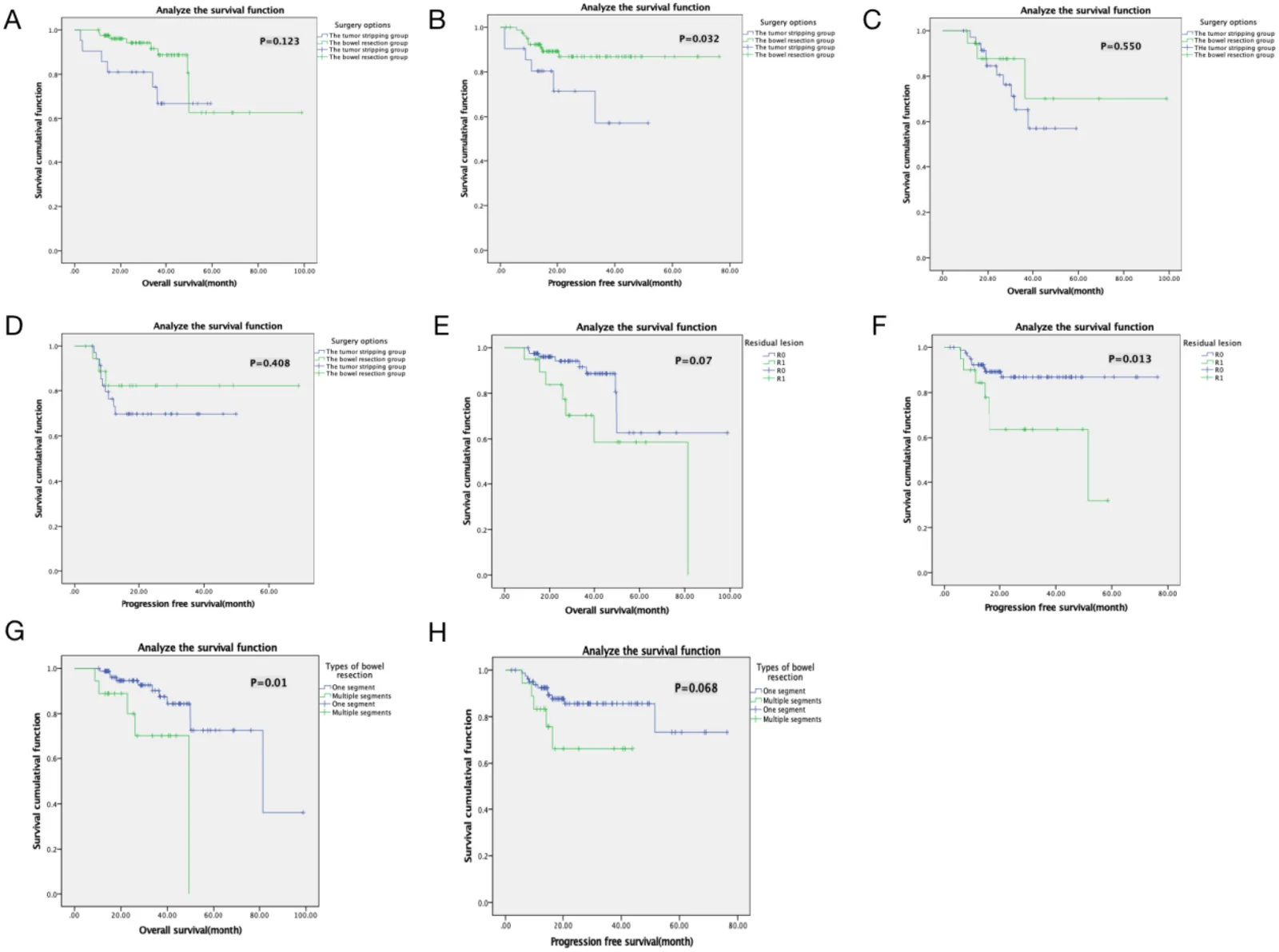
**(A)** Comparison of overall survival between the tumor stripping patients with only residual bowel wall lesion (R1) and the bowel resection patients with R0. **(B)** Comparison of progression free survival between the tumor stripping patients with only residual bowel wall lesion (R1) and the bowel resection patients with R0. **(C)** Comparison of overall survival between the bowel resection group and the tumor stripping group in IDS patients with R0. **(D)** Comparison of progression free survival between the bowel resection group and the tumor stripping group in IDS patients with R0. **(E)** Comparison of overall survival between R0 and R1 in the bowel resection group. **(F)** Comparison of progression free survival between R0 and R1 in the bowel resection group. **(G)** Comparison of overall survival between different types of bowel resection. **(H)** Comparison of progression free survival between different types of bowel resection.

In the tumor stripping group, 21 patients had only residual lesions confined to the bowel wall (R1). When comparing these 21 patients with the 81 patients in the bowel resection group who achieved R0, no significant difference in OS was observed (*P* = 0.123) (**Figure 2A**). However, PFS was significantly longer in the bowel resection group with R0 than in the tumor stripping group with isolated bowel wall residuals (R1) (*P* = 0.032) (**Figure 2B**). Among patients who underwent IDS and achieved R0, there were 19 in the bowel resection group and 36 in the tumor stripping group. In this subgroup, OS (*P* = 0.550) and PFS (*P* = 0.408) did not differ significantly between the two groups (**Figures 2C, D**). In the bowel resection group, 81 patients (80.20%) achieved R0 and 20 patients (19.80%) achieved R1. The 3−year OS of patients who achieved R0 was higher than that of patients with R1, but the difference was not statistically significant (*P* = 0.07) (**Figure 2E**). Patients who achieved R0 had significantly longer PFS than those with R1 (*P* = 0.013) (**Figure 2F**). Among the 101 patients in the bowel resection group, 84 (83.17%) underwent single−segment bowel resection and 17 (16.83%) underwent multisegment bowel resection. OS was significantly longer in patients with single−segment resection than in those with multisegment resection (*P* = 0.01) (**Figure 2G**). PFS did not differ significantly between the two groups (P = 0.068) (**Figure 2H**). Multivariable Cox regression analysis showed that neoadjuvant chemotherapy and residual disease size were independent factors affecting OS (**Table 5**). Pathological stage and neoadjuvant chemotherapy were independent factors affecting PFS (**Table 6**).

**Table 5:** Multivariate Cox regression analysis of factors related to OS.

	HR (95%CI)	*P* value
Age	0.468 (0.144-1.521)	0.207
Stage	1.870 (0.964-3.625)	0.064
III		
IV		
Cytoreductive Surgery	2.992 (1.598-5.602)	0.001*
PDS		
IDS		
Residual Disease	2.190 (1.058-4.533)	0.035*
R0		
R1 (<1cm)		
Bowel Surgery Type	0.969 (0.507-1.848)	0.923
Bowel Resection		
Bowel Stripping		

* P < 0.05, indicating a statistically significant difference.

**Table 6:** Multivariate Cox regression analysis of factors related to PFS.

	HR (95%CI)	*P* value
Age	0.480 (0.148-1.559)	0.222
Stage	2.057 (1.080-3.918)	0.028*
III		
IV		
Cytoreductive Surgery	2.937 (1.569-5.499)	0.001*
PDS		
IDS		
Residual Disease	2.014 (0.986-4.112)	0.055
R0		
R1 (<1cm)		
Surgical Type	0.936 (0.497-1.763)	0.839
Bowel Resection		
Bowel Stripping		

* P < 0.05, indicating a statistically significant difference.

## Discussion

Current evidence indicates that satisfactory cytoreductive surgery significantly improves prognosis in patients with advanced ovarian cancer. Specifically, each 10% increase in the extent of maximal cytoreduction (achieving no macroscopic residual disease) is associated with a 5.5% prolongation of median survival (12–15). In advanced ovarian cancer, bowel surgery is frequently required either during the primary procedure or later in the disease course for the management of recurrence or symptom palliation (1, 16). Gynecologic oncologists must carefully balance the risk of surgery−related complications against the potential oncologic benefits when determining the extent of resection.

Consistent with previous studies reporting that intestinal surgery increases the incidence of complications (17–20), this study further confirms that intestinal tumor resection significantly reduces the risk of intraoperative blood transfusion, postoperative pneumonia, and pleural effusion, without increasing the overall incidence of postoperative complications. The higher Surgical Complexity Score (SCS) in the intestinal resection group suggests that this procedure is more commonly performed in patients with more extensive disease, which may explain the relatively higher complication rate. Furthermore, the anastomotic fistula rate in the bowel resection group was 1.98%, consistent with the 0.8%–6.8% range reported in the gynecologic oncology literature (21–25), and approximately 26.17% of patients required ostomy creation, which is similar to results from previous studies.

The present study demonstrates that bowel tumor stripping is associated with a significantly reduced need for intraoperative red blood cell transfusion and lower incidences of postoperative pneumonia and pleural effusion, without increasing the overall rate of postoperative complications. In contrast, patients undergoing bowel resection had a higher overall complication rate. The higher SCS observed in the bowel resection group suggests that this procedure was more frequently performed in patients with more extensive disease, which may account for the elevated complication rates in this groupic finding consistent with previous reports that intestinal surgery increases complication rates. Moreover, the anastomotic leak rate in the bowel resection group was 1.98%, falling within the 0.8%ingon range reported in the gynecologic oncology literature. Approximately 26.17% of patients required stoma creation, a finding that aligns with earlier studies (21–25).

In agreement with earlier work suggesting that bowel resection increases the risk of surgical complications (17, 18), the present study demonstrates that bowel resection is associated with higher risks of infectious and respiratory complications. Furthermore, accumulating evidence indicates that extensive bowel resection adversely affects long−term bowel function and quality of life, with up to 40% of patients developing low anterior resection syndrome (LARS) following rectal surgery (26, 27). Although some scholars advocate aggressive bowel resection to maximize cytoreduction (28, 29), other studies suggest that extensive bowel resection should be avoided in the absence of a clear survival benefit, particularly given the risk of serious complications such as anastomotic leakage (25, 30). The findings of the present study align with the latter view, supporting that conservative tumor resection can achieve survival outcomes comparable to those of bowel resection while reducing perioperative trauma (7, 31).

In this study, we compared the therapeutic outcomes of bowel resection versus tumor stripping for intestinal metastases in patients undergoing surgery for advanced ovarian cancer. We found no evidence that tumor stripping adversely affected surgical complications or survival; OS and PFS were similar between the two groups. At 5 years, the OS rate was lower in the bowel resection group (65.8%) than in the tumor stripping group (73.8%), and the 5−year PFS rate was also lower in the bowel resection group (70.6%) than in the tumor stripping group (80.3%). In subgroup analyses restricted to patients who achieved R0 resection, no significant differences in OS or PFS were observed between the two groups. Similarly, among patients with R1 resection, OS and PFS were comparable between the two groups. Of note, multivariable Cox regression analysis revealed that pathological stage, administration of neoadjuvant chemotherapy, and residual lesion size were significant independent prognostic factors. Consistent with our findings, previous studies have reported that intestinal involvement is associated with poor prognosis, but that bowel resection does not confer a survival benefit in patients with advanced ovarian cancer, who undergo more extensive radical surgery without prolongation of survival (1, 28).

We further analyzed patients within the bowel resection group and found that the 5−year survival rate of patients who achieved R0 (62.7%) was similar to that of patients with R1 (58.6%), with the difference approaching statistical significance (*P* near 0.05). Patients in the R0 bowel resection group had significantly longer PFS than those in the R1 bowel resection group. We also performed a survival comparison between patients in the tumor stripping group who had residual disease confined to the bowel wall (R1) and patients in the bowel resection group who achieved R0. OS was similar between these two subgroups, a finding that may be influenced by limitations such as retrospective study bias and small sample size. However, patients in the bowel resection (R0) group had significantly longer PFS than those in the tumor stripping (R1) group with isolated bowel wall residuals. According to patient records, the primary reason for the presence of residual disease confined to the bowel wall was patient or family refusal of bowel resection. In such circumstances, a careful risk−benefit assessment is warranted. When complete resection can be achieved by bowel resection, the procedure is recommended to delay disease recurrence. Therefore, bowel resection should be considered only when complete cytoreduction can be achieved or when it is necessary to alleviate or prevent intestinal obstruction.

Numerous studies have investigated the role of NACT in reducing tumor burden, which may lower the rate of bowel resection—a procedure performed in 40–80% of patients undergoing PDS (32, 33). In contrast, bowel resection rates of 8–49% have been reported in patients undergoing IDS (10, 34–36). In the present study, the bowel resection rates were 41.67% in the PDS group and 34.67% in the IDS group. McNamara et al. reported that the number of preoperative chemotherapy cycles did not affect the bowel resection rate. Among patients with advanced ovarian cancer undergoing IDS, those who required bowel resection had an increased 3−year risk of death, with no impact on progression−free survival (37). The present study similarly found that the number of preoperative chemotherapy cycles did not influence the bowel resection rate. For many patients receiving NACT, bowel resection remains necessary to achieve satisfactory cytoreductive outcomes. In patients with advanced ovarian cancer and intestinal metastases, the depth of bowel wall invasion or the size of the metastatic lesion may change after NACT. Therefore, gynecologic oncologists must carefully assess the optimal surgical approach for managing intestinal metastases intraoperatively.

A key strength of this study is the consecutive enrollment of patients, which minimizes selection bias. However, several limitations should be acknowledged. First, as a single−center, retrospective study, it is subject to selection, indication, and confounding biases. Assignment to bowel resection or tumor stripping was based on intraoperative findings, surgeon judgment, and patient preference, not random allocation, potentially causing baseline imbalances. Second, small subgroup sizes (e.g., multisegment resection or R1 with isolated bowel wall residuals) reduce statistical power and affect survival estimate stability. Third, the retrospective design precluded systematic collection of long−term quality−of−life data, including key intestinal function endpoints such as low anterior resection syndrome and stoma−related outcomes. Fourth, single−center findings may not generalize to centers with different surgical volumes or protocols. Thus, results should be interpreted cautiously. Future prospective, multicenter, controlled studies with standardized protocols and comprehensive follow−up are needed to validate oncologic and functional outcomes of tumor stripping versus bowel resection. Meanwhile, the clinical phenotypes and gene expression profiles of resected intestinal metastases warrant further investigation to guide treatment.

Despite these limitations, our findings support a practical intraoperative strategy for patients with advanced epithelial ovarian cancer and intestinal metastases. When R0 cytoreduction is deemed achievable, intestinal tumor stripping should be prioritized, as it reduces surgical trauma and perioperative complications without compromising oncologic outcomes. Bowel resection should be reserved for cases where tumor invasion is too deep for complete removal by stripping alone, and extensive bowel resection should be avoided whenever possible. This individualized, stepwise approach helps balance surgical safety, postoperative quality of life, and survival benefit. Prospective studies are warranted to validate this strategy across different centers.

## Conclusion

Bowel tumor stripping does not negatively affect the prognosis of advanced ovarian cancer if R0 cytoreductive surgery is achieved. Excessive bowel resection does not improve overall survival.

## Data Availability

The original contributions presented in the study are included in the article/supplementary material. Further inquiries can be directed to the corresponding author.
