# Correction: Surgical treatment and prognosis in patients with intestinal metastases originated from advanced epithelial ovarian cancer

**DOI:** 10.3389/fonc.2026.1933683

**Published:** 2026-08-13

**Authors:** Hongxia Wang, Yijie Li, Zhifen Yang, Jinxiu Wang, Kaiyun Qin, Yu Yu, Na Wang, Jingde Jia, Wenhong Zhao, Fenghua Zhang, Mario M. Leitao, Ran Meng, Yueping Liu, Yan Ding, Zhengmao Zhang

**Affiliations:** 1 Department of Gynecology, Fourth Hospital of Hebei Medical University, Shijiazhuang, Hebei, China; 2 Department of Obstetrics, Fourth Hospital of Hebei Medical University, Shijiazhuang, Hebei, China; 3 Department of Gynecology, Hebei General Hospital, Shijiazhuang, Hebei, China; 4 Department of General Surgery, Hebei General Hospital, Shijiazhuang, Hebei, China; 5 Gynecology Service, Department of Surgery, Memorial Sloan Kettering Cancer Center, New York, NY, United States; 6 Department of Pathology, Fourth Hospital of Hebei Medical University, Shijiazhuang, Hebei, China

**Keywords:** advanced ovarian cancer, intestinal metastasis, intestinal resection, intestinal tumor stripping, prognosis, surgical treatment

There was a mistake in **Table 1** as published. There is an error in **Table 1** regarding the FIGO Stages (2014) for Stages IA and IIB. The formatting of the term “Other” in **Table 1** is incorrect. The correct terms should be Stages “IIIA and IIIB”, and the formatting of the term “Other” should be consistent with that of “Endometrioid”. The corrected **Table 1** appears below.

**Table 1:** Preoperative basic characteristic data of ovarian cancer patients.

Characteristics	Bowel resection group (n=101)	Bowel tumor stripping group (n=154)	P value
Age (y)	56.24 ± 9.51	54.90 ± 9.15	0.261
FIGO Stage (2014)			0.021*
IIIA	5 (4.95)	10 (6.49)	
IIIB	5 (4.95)	20 (12.99)	
IIIC	70 (69.31)	108 (70.13)	
IV	21 (20.79)	16 (10.39)	
Pathology			0.097
Endometrioid	4 (4.0)	18 (11.7)	
Mucinous	1 (1.0)	2 (1.3)	
Serous	88 (87.1)	114 (74.0)	
High grade	86 (97.73)	109 (95.61)	
Low grade	2 (2.27)	5 (4.39)	
Clear Cell	0 (0)	1 (0.6)	
Mixed	2 (2.0)	6 (3.1)	
Serous Cystadenocarcinoma	3 (3.0)	2 (2.0)	
Others	3 (3.0)	11 (7.1)	
Hospital Stays (d)	22.77 ± 9.54	19.40 ± 5.77	0.001*
Surgical Type			0.298
PDS	75 (74.26)	105 (68.18)	
IDS	26 (25.74)	49 (31.82)	
NACT Cycles			0.521
1-3	19 (73.08)	39 (79.59)	
≥4	7 (26.92)	10 (20.41)	

*P < 0.05, indicating a statistically significant difference.

A correction has been made to the section **Materials and methods**, “*Patients*”, Paragraph 5. The phrase “3orksycles” in this paragraph contains an error; the correct wording is “3–4 cycles”:

“All patients received a liquid diet starting 3 days before surgery for bowel preparation and took oral laxatives 1 day preoperatively. According to current guidelines, NACT was indicated for patients with advanced epithelial ovarian cancer who were not candidates for immediate surgery due to extensive disease and high perioperative risk. NACT consisted of platinum-based combination chemotherapy, typically paclitaxel plus carboplatin, administered every 3 weeks for 3–4 cycles. Patients who underwent interval debulking surgery (IDS) and achieved R0 resection received at least 3 cycles of adjuvant chemotherapy postoperatively. Patients who underwent primary debulking surgery (PDS) received postoperative platinum-based first-line chemotherapy, either alone or in combination with paclitaxel, every 3–4 weeks for 6–8 cycles. The follow-up period for all patients ranged from 2 to 98 months”.

The original version of this article has been updated.

